# Taurolidine reduces the tumor stimulating cytokine interleukin-1beta in patients with resectable gastrointestinal cancer: a multicentre prospective randomized trial

**DOI:** 10.1186/1477-7819-7-32

**Published:** 2009-03-23

**Authors:** Chris Braumann, Carsten N Gutt, Johannes Scheele, Charalambos Menenakos, Wilhelm Willems, Joachim M Mueller, Christoph A Jacobi

**Affiliations:** 1 Department of General, Visceral, Vascular and Thoracic Surgery, Universitaetsmedizin Berlin, Charité Campus Mitte, Humboldt University, Charitéplatz 1, 10117 Berlin, Germany; 2 Division of Molecular Biology, Universitaetsmedizin Berlin, Charité Campus Mitte, Humboldt University, Charitéplatz 1, 10117 Berlin, Germany; 3 Department of Surgery, Johann Wolfgang Goethe-University Hospital, Frankfurt am Main, Germany; 4 Department of General and Visceral Surgery, Friedrich-Schiller-University, Jena, Germany

## Abstract

**Background:**

The effect of additional treatment strategies with antineoplastic agents on intraperitoneal tumor stimulating interleukin levels are unclear. Taurolidine and Povidone-iodine have been mainly used for abdominal lavage in Germany and Europe.

**Methods:**

In the settings of a multicentre (three University Hospitals) prospective randomized controlled trial 120 patients were randomly allocated to receive either 0.5% taurolidine/2,500 IU heparin (TRD) or 0.25% povidone-iodine (control) intraperitoneally for resectable colorectal, gastric or pancreatic cancers. Due to the fact that IL-1beta (produced by macrophages) is preoperatively indifferent in various gastrointestinal cancer types our major outcome criterion was the perioperative (overall) level of IL-1beta in peritoneal fluid.

**Results:**

Cytokine values were significantly lower after TRD lavage for IL-1beta, IL-6, and IL-10. Perioperative complications did not differ. The median follow-up was 50.0 months. The overall mortality rate (28 vs. 25, p = 0.36), the cancer-related death rate (17 vs. 19, p = .2), the local recurrence rate (7 vs. 12, p = .16), the distant metastasis rate (13 vs. 18, p = 0.2) as well as the time to relapse were not statistically significant different.

**Conclusion:**

Reduced cytokine levels might explain a short term antitumorigenic intraperitoneal effect of TRD. But, this study analyzed different types of cancer. Therefore, we set up a multicentre randomized trial in patients undergoing curative colorectal cancer resection.

**Trial registration:**

ISRCTN66478538

## Background

Surgery remains the only therapeutic treatment with cure as a possible outcome for patients with colorectal, gastric or pancreatic cancer. During resection of gastrointestinal malignancies tumor cells can be released and spread. This leads to local recurrence, peritoneal carcinomatosis or distant metastases with poor prognosis [1,2]. Despite improvements in surgical techniques and use of recent chemotherapy agents and radiotherapy protocols, most patients with gastric or pancreatic cancer ultimately die of their disease. Therefore intraoperative intraperitoneal lavage with antitumorigenic solutions may be a valuable new strategy for gastrointestinal malignancies.

The antineoplastic substance taurolidine (TRD) has been proved to suppress intraperitoneal gastrointestinal tumor growth after laparotomy and laparoscopy in animals [3-5]. TRD has no relevant side effects on haematopoiesis, liver or renal function in animals. It induces apoptosis and inhibits growth of various tumor cell lines *in vitro *[6-8]. TRD reduces pancreatic cancer progression [9] as well as TNF-α and VEGF secretion by gastrointestinal neoplasms due to the inhibition of protein biosynthesis in tumor cells [10]. The substance is found under US Patent 7151099. Another interesting substance, heparin, binds to extracellular receptors and blocks adhesion of tumor cells to the peritoneal surface [11]. No relevant side effects have been described with the concentration we used intraoperatively. Both substances have been previously used.

Povidone-iodine is a substance commonly used by many institutions in Germany in peritoneal lavages to prevent wound infections (and tumor growth) [12]. There are presently no clinical studies investigating the role of povidone-iodine in tumor growth after tumor resection. In an toxicity study, more than 0.16% of povidone-iodine was antitumorigenic in almost all tumor cells tested and showed severe cytotoxicity *in vivo *and *in vitro *[13]. Nevertheless, resorption of povidone-iodine, particularly the PVP-17-molecule, is still unknown and its metabolism in the liver seems to be problematic. This substance reduces local immune response due to damaged peritoneal macrophages and mesothelial cells. It enhances fibrin production on the liver surface and induces adhesions [11]. Radical surgical treatment of gastrointestinal cancer can be potentially improved by the intraperitoneal administration of agents such as TRD or povidone-iodine. However, their impact on cytokine release, toxicity, loco-regional recurrence, distant metastases, and survival time remains unclear.

This study aimed to assess these substances when instilled intraoperatively before and after tumor resection. Intraperitoneal and serum levels of different cytokines and inflammatory markers were evaluated perioperatively. Morbidity, mortality, follow-up, and survival rates were consecutively assessed.

## Methods

### Patients

Inclusion criteria to the multicentre prospective randomized controlled trial were open resection of the colon, gastric or pancreatic cancer, a physical status classified as ASA I-III, and histopathological R0-resection. Exclusion criteria were complete ileus, a physical status classified as ASA IV, histopathological R1- or R2-resection, unknown metastases detected intraoperativaly, peritoneal carcinomatosis, intraabdominal abscess, sepsis or clinical relevant organ failure, and apparent coagulopathy.

The trial was set up on a multicentre level in three university hospitals in Germany. Between January 1999 and August 2001 we included adults undergoing elective conventional colectomies, gastrectomies or pancreatectomies due to cancer. AJCC/UICC TNM classification and stage groupings were used.

Patients were admitted to the study in accordance to the inclusion criteria after receiving detailed information about the trial orally and in a written form and after having given their written consent for the participation in the study. The study was approved by ethics committee.

### Procedures and Monitoring

A local investigator in each centre was responsible for patient admission to the study. All patients were enrolled strictly to the protocol. Randomisation was based on the computer program "RANDOMA" one day before surgery. The operation team was informed of the intraperitoneal treatment regime after opening the abdomen. The operations were performed by three surgeons in each centre (total of nine), all of them with excellent command of oncologic surgical principles. Each surgeon operated patients with all three types of cancer.

**Figure 1:**
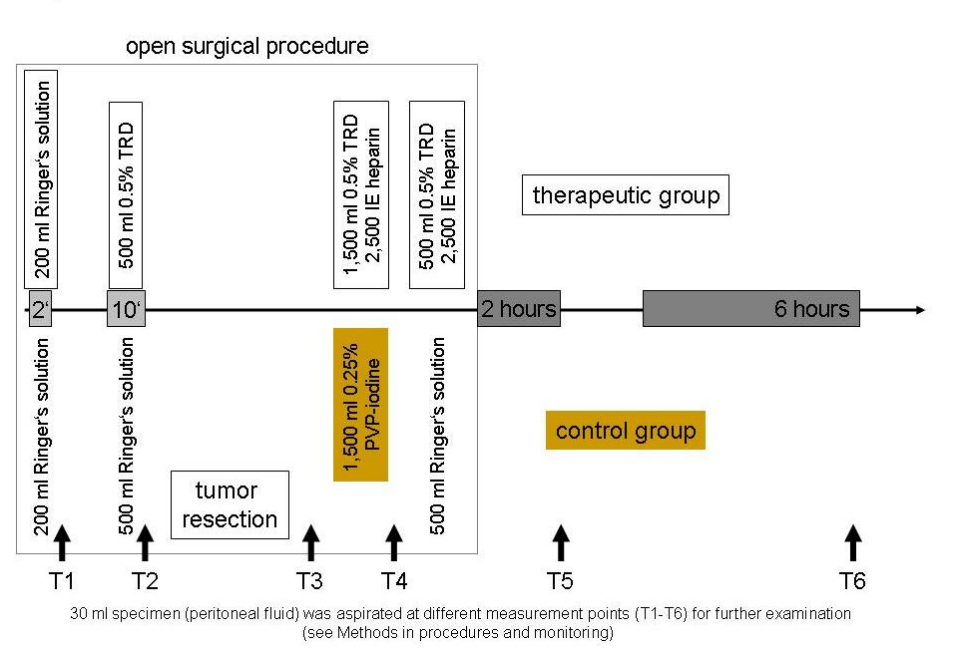
**Schematic diagram illustrating the study protocol**.

The patients were randomized into TRD group (n = 60) or povidone-iodine group (control group, n = 60). All operations were performed conventionally. Immediately after opening the abdomen, 200 ml of Ringer's solution was instilled into the peritoneal cavity for 2 minutes in both groups. 30 ml of peritoneal fluid was collected (measurement time T1) to evaluate different cytokine levels (IL-1β, IL-6, and IL-10). After removal of the residual fluid the therapeutic substances were administered intraperitoneally and left for 10 minutes: either 500 ml of 0.5% TRD solution or 500 ml of Ringer's solution (control group). Again 30 ml of peritoneal fluid was aspirated and analyzed (T2). A subsequent tumor resection was then carried out according to the principles of surgical oncology. 30 ml of peritoneal fluid was then absorbed (measurement T3). At the end of the operation patients underwent a second therapeutic peritoneal irrigation for 10 minutes: either 1,500 ml 0.5% TRD with 2,500 IU heparin (TRD group) or 1,500 ml 0.25% povidone-iodine solution (control group). Again 30 ml of fluid was removed and analyzed (T4). Before the incision was closed in layers, either 500 ml of 0.5% TRD with 2,500 IU heparin (TRD group) or 500 ml of Ringer's solution (control group) were instilled. One drainage was placed in Douglas' space and barred in all patients. 2 hours (h) and 6 h after the operation 30 ml of peritoneal fluid were taken from the drainage in order to measure T5 and T6, respectively (Figure 1). Samples were any time identical diluted, immediately cooled (4° Celsius; C), centrifuged, and supernatants were stored (-25°C). The remaining of the intraperitoneal fluid was collected to determine intraabdominal viable tumor cells (in T1 and T4). The volume of the removed material was 30 ml in all measurements and standardized aspiration after insertion of the same quantity either of TRD or Ringer's solution permitted a balanced aspiration from an equal dilution in all cases. Central venous blood was also taken at the same assessment points for the determination of the cytokine levels. Peri- and postoperative coagulation status, and peritoneal and serum cytokine concentrations (IL-1β, IL-6, and IL-10) were investigated.

Specimen for the measurement of the Interleukins were collected and preserved as recommended by the manufacturer.

### Interleukin 1-beta measurement

This kit was especially develeoped for the quantitative determination of human interleukin 1 beta (IL-1beta) Immunoassay concentrations in cell culture supernates, serum, and plasma. The parameters were determined in duplicates by enzyme linked immunosorbent assay (ELISA) technique as recommended by the manufacturer R&D Systems GmbH Wiesbaden-Nordenstadt, Germany (sensitivity 4.9–2,000 pg/ml, Catalog Number OLB00B). Cell culture supernate samples may require dilution to read on the standard curve. If samples are suspected to be more than 1000 pg/mL, specimens were diluted in Calibrator Diluent RD6-10. No samples of IL-1beta required larger dilutions. Probes were examined as recommended by the manufacturer.

### Interleukin 6 and 8

The Quantikine HS human IL-6 Immunoassay and the IL-6 Immunoassay were developed for the quantitative determination of human interleukin concentrations in serum, plasma (, and urine). Probes were examined as recommended by the manufacturer.

Blood samples were also taken preoperatively and on days 1, 2, 4, and 7 postoperatively for the determination of leukocyte counts, HLA-DR expression, and C-reactive protein levels in both groups.

### Clinical management

Patients were admitted under the care of one visceral consultant surgeon per centre. Preoperatively, patients were given detailed information of the procedure. All patients underwent identical bowel preparation one day before surgery. Patients were allowed to drink fluids up to 4 h before the operation. Anaesthesiologists were informed of the details of the study. Before induction of general anaesthesia a single dose of antibiotic prophylaxis with cefuroxim (1.5 g iv) and metronidazol (500 mg iv) was given. A urinary catheter was placed transurethrically in all patients. Clinical decisions about hospital discharge were made by the treating surgical team and not by the investigators. In case of a colectomy a midline laparotomy was carried out. For a gastrectomy and pancreatectomy a transverse sub-costal incision of the abdomen was performed. Postoperative pain was managed by patient-controlled analgesia devices delivering intravenous morphine.

### Therapeutic agents

Abdominal irrigations/dilutions were identical at any time point.

TRD (Taurolin^®^) was applied in a 0.5% solution (purchased from Geistlich Pharma AG, Wohlhusen, Switzerland). At the end of the operation TRD and 2,500 IU heparin were instilled.

Although it has been clearly demonstrated that Povidone-iodine (Braunol^®^) should be avoided for abdominal lavage, many German clinics use the diluted agent in peritonitis/sepsis and after colonic or rectal tumor resection. It was purchased from B. Braun Melsungen AG (Melsungen, Germany) and was applied intraperitoneally in a 0.25% solution.

### Postoperative course

Patient follow-up was carried out according to a standardized protocol. After hospital treatment, all the patients were followed-up on an outpatients' basis. Postoperatively, patients were thoroughly examined on the 1, 2, and 3 months, and every 3 months thereafter. Follow-up included physical examination, blood counts and biochemistry, ultrasound of the abdomen, and in suspicion of relapse, abdominal CT-scan. Pulmonary metastases were detected with thoracic X-ray or thoracic CT-scan. Results of a median follow-up of 50.0 months (range 1.7–72.6) are currently reported.

### Ethical and Human Considerations

The Ethics Committee of the Charité Campus Mitte, University Hospital, Universitaetsmedizin Berlin, Germany, was the leading study centre and approved the study design. The study was carried out in accord with the ethical standards of the Helsinki declaration of 1975. Written informed consent was obtained from all patients before enrolment. The study has been registered in the International Standard Randomized Controlled Trial Number Register (ISRCTN66478538).

### Sample size, power calculation, and statistical analysis

The main endpoint of the study was the determination of the perioperative concentration of IL-1β in the patient's peritoneal fluid. On the basis of Badia *et al*. [14] we assumed that the intraperitoneal concentration of IL-1β (190.6 ± 90 pg/ml) eight hours after a pancreas operation is at least 30% lower in the taurolidine group compared to the povidone-iodine group. For a 0.05 difference with a power of 80% every group included 60 patients. We also assessed other long-term values: probability of overall survival and of being free of recurrence. Because of the objective of the study patients with metastatic disease were excluded. Time to metastasize, local recurrence or death was calculated from surgical resection to the last visit, call or death. For cancer-related survival, patients who died from other causes were evaluated at time of death. Survival, metastases, and recurrence rates were calculated with the Kaplan-Meier method and differences were tested by log-rank test. Categorical variables were compared by chi-squared test (Fisher's exact test). Continuous variables were evaluated by the Student-t-test or Mann-Whitney-U-test, depending on the distribution. Results were considered significant if the two-sided p values were 0.05 or less. Tests were performed with the statistical software SPSS 14.0 for Windows. An independent data monitoring committee was appointed to review all data.

## Results

**Figure 2:**
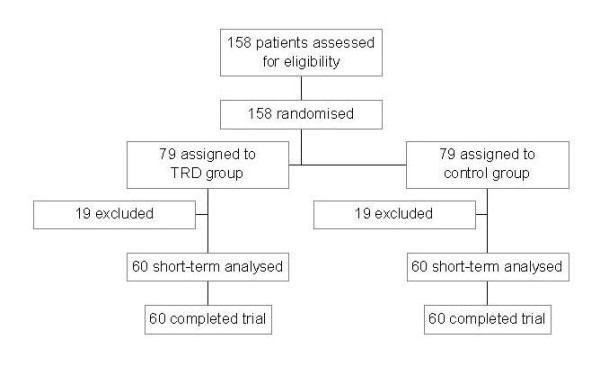
**Trial profile**.

From the 158 patients assessed for eligibility for the study, 38 were excluded. Patients enrolled in the study included for TRD: Berlin n = 25, Frankfurt n = 18, Jena n = 17, and for povidone-iodine: Berlin n = 24, Frankfurt n = 18, and Jena n = 18. Figure 2 shows the trial profile. 120 patients took part in the study, with 60 patients in each group. The first operation was performed in February 2001 (follow-up 72 months) and the last organ resection was performed in August 2003 (42 months), so that the follow-up for all patients was at the end of 2007. The data were interpreted and the statistical analysis was performed in 2008. Detailed characteristics of patients including demographic profiles, ASA classification, co-morbidities, and the type of operation performed did not differ between the groups and are listed in Table 1. Types and sites of disease including the Union International Contre Le Cancer classifications (UICC) are also listed in Table 1.

**Table 1:** Patient's baseline and clinical characteristics

	**Taurolidine group** **n = 60**		**Control group n = 60**		**p**
**Age (years)**	66.9 (26.4–87.1)		64.5 (22.8–80.8)		0.08

**Sex (male/female)**	37 (61.7%)/23 (38.3%)		35 (58.3%)/25 (41.7%)		-

**Height**	170 (7.7)		171 (8.8)		0.76

**Weight**	74.2 (13.7)		74.2 (11.8)		0.92

**Type of cancer**		**n = 60**		**n = 60**	
Colon	48.3%	29	46.7%	28	
Gastric	43.3%	26	43.3%	26	
Pancreatic	8.3%	5	10%	6	

**UICC histopathological staging**					
**Colon cancer**		**29**		**28**	
I		8		9	
II		8		9	
III		13		10	
**Gastric cancer**		**26**		**26**	
IA		4		5	
IB		6		7	
II		8		5	
IIIA		5		6	
IIIB		3		3	
**Pancreatic cancer**		**5**		**6**	
I				2	
II		2		2	
III		3		2	

**ASA classification (all patients)**					
I	23.3%	14	23.3%	14	
II	38.3%	23	38.3%	23	
III	38.3%	23	38.3%	23	

**Risk factors**		**32**		**24**	
Arterial hypertension	33.3%	20	26.6%	16	
Chronic obstructive lung disease	1.6%	1	5%	3	
Diabetes mellitus	13.3%	8	6.6%	4	
Nephropathy	5%	3	1.6%	1	

**Tumour location**					
**Colon**:					
Caecum				1	
Ascending colon		5		3	
Transverse colon		3		6	
Descending colon		2		3	
Sigmoid colon		5		5	
Rectum		14		10	
**Stomach**:					
Gastric cardia		5		3	
Gastric fundus		4		6	
Gastric body		6		9	
Gastric antrum		11		8	
**Pancreas**:					
Pancreatic head		5		6	

**Intervention**					
Right colectomy		4		4	
Extended right colectomy		2		1	
Left colectomy		2		6	
Extended left colectomy		1		2	
Sigmoidectomy		5		4	
Rectum resection		10		9	
Abdominoperineal resection		4		1	
Subtotal colectomy		1		1	
Total gastrectomy		14		19	
Total gastrectomy + splenectomy		3		2	
Subtotal (3/4) gastrectomy		8		5	
Distal esophageal resection		1			
Pancreatic head resection (Kausch-Whipple' resection)		1		3	
Pylorus-preserving duodenopancreatectomy		4		3	

Values are median (range), mean (standard deviation) or number, esophagectomy: adenocarcinoma of the cardia (Type Siewert I)

Risk factors, particularly diabetes mellitus, were higher in the TRD group (Table 1).

**Figure 3:**
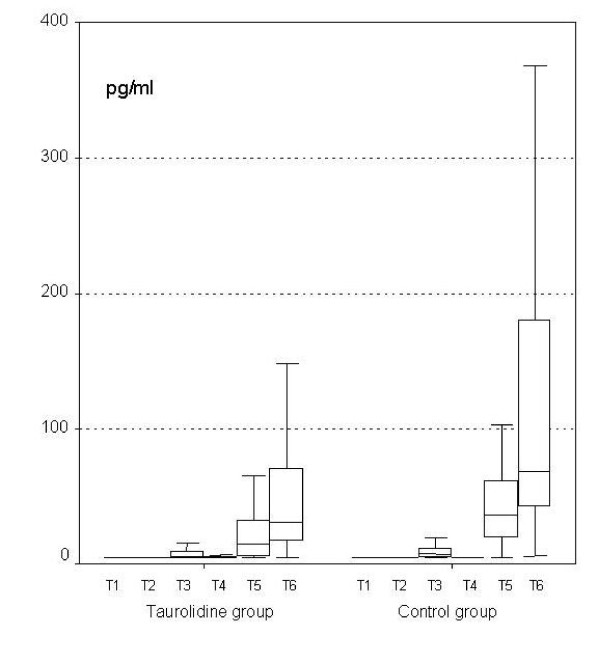
**Perioperative IL-1β levels of the peritoneal fluid**.

We were interested in the effects of the used intraoperative irrigation fluid on morbidity, mortality rates, as well as on perioperative complications. Therefore, the overall rates are shown in Table 2. In intraperitoneal samples IL-1β values were lower in the TRD group (shown in Figure 3; T3 measurement p = 0.029, T5 p < 0.001, T6 p < 0.001). Similar data were obtained with IL-6 (shown in Table 3a; T3 p = 0.048, T4 p = 0.017, T5 p = 0.003, and T6 p = 0.008), and with IL-10 (in T5, p < 0.001 and T6, p < 0.001). There was no statistically significant difference between other Interleukin values in both groups in the intraperitoneal fluid. No viable tumor cells were detected in T1–T3 situations. T4 sample cytology was positive for malignant cells in only one patient from each group.

**Table 2:** Data related to surgical intervention

	**Taurolidine group** **(n = 60)**	**Control group** **(n = 60)**	**p**
**Duration of intervention (min)**			
Colon	206 (74)	191 (53)	0.39
Stomach	259 (57)	272 (102)	0.57
Pancreas	318 (58)	371 (163)	0.51

**Perioperative mortality rate**	**2**	**3**	**0.5**
Colon		1	0.5
Stomach	2	1	0.5
Pancreas		1	0.5

**Perioperative morbidity rate**	**17**	**20**	**0.35**
Colon	8	6	0.54
Stomach	8	9	0.5
Pancreas		3	0.12
**Surgery-related complications**	**11**	**13**	**0.41**
Anastomotic leak	6	6	-
Peritonitis	5	3	0.36
Intraperitoneal hemorrhage	1		-
Subcutaneous wound infection	5	11	0.08
**Non-surgery complications**	**12**	**13**	**0.5**
Myocardial infarction	1	2	0.5
Transient cardiac arrhythmia	4	6	0.37
Pneumonia/pleural effusion	9	10	0.5
Acute renal failure	1	3	0.31
Hepatic decompensation		1	0.5

Data are mean (SD), median* (range) or number of patients; 30 days mortaility- and complication rate: chi-squared test (Fisher's exact)

**Table 3:** Cytokine levels of peritoneal fluid and serum (pg/ml); tumor cell detection; taurolidine group (TRD); povidone-iodine group (C).

	**T1**		**T2**		**T3**		**T4**		**T5**		**T6**	
**Peritoneal fluid**	**TRD**	**C**	**TRD**	**C**	**TRD**	**C**	**TRD**	**C**	**TRD**	**C**	**TRD**	**C**

IL-1β *	4.9 (4.9–4.9)	4.9 (4.9–6.2)	4.9 (4.9–4.9)	4.9 (4.9–9.7)	**4.9** **(4.9–58.4)**	**7.5** **(4.9–129)**	4.9 (4.9–41.8)	4.9 (4.9–54.0)	**15.0** **(4.9–441)**	**36.6** **(4.9–561)**	**31.1** **(4.9–1410)**	**68.8** **(6.3–1001)**

IL-6 *	4.9 (4.9–325)	4.9 (4.9–216)	4.9 (4.9–197)	4.9 (4.9–568)	**429** **(4.9–5350)**	**738** **(4.9–10000)**	**261** **(4.9–6580)**	**455** **(4.9–20000)**	**4530** **(26–10000)**	**5001** **(273–20000)**	**5001** **(7–10000)**	**5700** **(1001–20000)**

IL-10 *	1.6 (0.8–50.5)	1.7 (1.0–21.3)	1.6 (0.8–36.2)	1.6 (0.9–13.7)	5.6 (0.8–81.4)	9.0 (1.6–98.8)	4.1 (1.1–31.4)	4.0 (1.6–87.6)	**27.2** **(0.8–100)**	**61.5** **(3.0–250)**	**39.2** **(1.6–155)**	**88.8** **(4.0–250)**

Tumour cells	0	0					1 (1.7%)	1 (1.7%)				

**Serum**												

IL-1β												

IL-6 *	4.9 (4.9–17.0)	4.9 (4.9–29.8)	4.9 (4.9–49.8)	4.9 (4.9–83.8)	43.0 (4.9–326)	42.6 (4.9–302)	43.5 (4.9–288)	44.3 (4.9–553)	**87.7** **(11–1500)**	**138** **(10–650)**	**83** **(4.9–1480)**	**165** **(13–3460)**

IL-10 *	**1.6** **(0.8–25.0)**	**1.7** **(0.8–38.8)**	1.6 (0.8–25.0)	1.6 (0.8–38.8)	4.7 (1.6–100)	6.1 (0.8–108)	4.6 (1.6–77)	7.9 (1.6–135)	6.5 (1.6–85)	9.1 (1.5–248)	7.0 (1.6–50.0)	6.9 (1.6–223)

Values are given as mean (SD), t-test or median* (range), Mann-Whitney-U-test; statistical significance is shown in bold letters

In serum samples IL-1β was not detectable (Table 3). IL-6 levels were lower in the TRD group (T5 p = 0.012, T6 p = 0.009) and serum IL-10 of the TRD group was lower in T1 (p = 0.026). Monocyte HLA-DR level suppression was analyzed on postoperative day 2 (Table 4; p < 0.05). No statistically significant differences were observed in the CRP values.

**Table 4:** Biochemistry

	**Preop**		**Postop day 1**		**Day 2**		**Day 4**		**Day 7**	
**Blood parameters**	**TRD**	**C**	**TRD**	**C**	**TRD**	**C**	**TRD**	**C**	**TRD**	**C**

Leukocytes (/μl)	7.2 (2.4)	6.6 (2.6)	10.4 (3.2)	10.9 (4.1)	10.9 (3.7)	11.2 (3.2)	8.2 (2.6)	8.5 (3.3)	9.7 (3.3)	10.2 (5.0)

HLA-DR4 (monocyte expression)	32.3	31.5	**38.1**	**30.5**	**46.1**	**28.6**	37.5	34.9	49.1	51.2

CRP (mg/dl)	1.5 (3.0)	1.0 (2.1)	8.4 (3.9)	8.5 (3.7)	13.2 (6.8)	13.6 (5.5)	10.0 (8.2)	9.5 (6.4)	8.3 (7.7)	7.2 (6.8)

Values are given as mean (SD), t-test or median* (range) in pg/ml, Mann-Whitney-U-test; statistical significance is shown in bold letters

Patients with clinical relevant peritonitis were treated on ICU whereas patients with subcutaneous wound infections were discharged. The mortality rate at 30 days postoperative was 3.3% (2 patients) and 5% (3 patients) in the TRD group and in the control group, respectively. The difference was statistically insignificant. The median length of follow-up is shown in Table 5. The focus of this study was not the use of the agents in a special tumor type-we paid attention on the effects of macrophage-produced IL-1 beta in different gastrointestinal tumors. After surgery, 14 patients of the TRD group (23%) and 21 patients of the control group (33%) received adjuvant chemotherapy and radiotherapy (9 vs. 7) according to the established protocol. Although less patients in the TRD group received chemotherapy, especially with gastric cancer (p = 0.02), no effects on overall tumor recurrence rate were detected (p = 0.35), neither in time to recurrence (p = 0.59) nor time to metastasis (p = 0.24). The loco-regional recurrence rate (p = 0.16), the rate of distant metastases (p = 0.2), and the cancer-related mortality did not differ between the groups (Table 4). Data on probability of staying free of relapse are not shown.

**Table 5:** Tumor recurrence and mortality

	**Taurolidine group** **(n = 60)**	**Control group** **(n = 60)**	**P**
**Adjuvant chemotherapy**	**14**	**21**	**.11**
Colon cancer	11	10	-
Gastric cancer	1	7	0.02
Pancreatic cancer	2	4	-

**Adjuvant radiotherapy**	**9**	**7**	**0.39**
Colon cancer	7	6	-
Gastric cancer	2	1	-
Pancreatic cancer	-	-	-

Follow-up* (months)	**50.0 (1.7–72.0)**		
Colon cancer	53.5 (9.0–72.1)	59.2 (19.6–72.0)	0.41
Gastric cancer	39.9 (1.7–72.0)	43.7 (5.7–67.3)	0.84
Pancreatic cancer	15.6 (8.5–34.5)	10.7 (8.8–46.9)	0.66

**Overall tumour recurrence rate**	**19**	**22**	**0.35**

**Type of recurrence**			

**Loco-regional recurrence rate**	**7**	**12**	**0.16**
Colon cancer	2	4	0.32
Gastric cancer	3	5	0.35
Pancreatic cancer	2	3	0.61

Time to local recurrence* (months)	**17.8 (5.6–35.6)**	**16.0 (5.3–26.5)**	**0.43**
Colon cancer	10.9 and 35.6	18.4 (6.0)	1
Gastric cancer	18.2 (2.9)	14.9 (4.4)	0.39
Pancreatic cancer	5.6 and 21.3	10.8 (7.0)	0.8

**Distant metastasis rate**	**13**	**18**	**0.2**
Colon cancer	6	8	0.35
Gastric cancer	4	6	0.36
Pancreatic cancer	3	4	0.65

Time to metastasis * (months)	**21.0 (5.2–44.5)**	**14.4 (3.0–35.9)**	**0.17**
Colon cancer	23.7 (11.4–44.5)	19.3 (3.0–35.9)	0.28
Gastric cancer	16.5 (5.2–34.1)	14.0 (5.9–24.1)	0.76
Pancreatic cancer	22.3 (12.3–24.0)	12.3 (4.0–18.6)	0.23

**Overall mortality rate**	**28**	**25**	**0.36**
Colon cancer	8	7	0.53
Gastric cancer	15	12	0.29
Pancreatic cancer	5	6	-

Time to death * (months)	**20.0 (0.1–61.0)**	**19.6 (0.1–58.5)**	**0.71**
Colon cancer	35.6 (9.0–61.0)	22.1 (0.1–58.5)	0.34
Gastric cancer	12.8 (0.1–58.6)	12.4 (0.8–40.1)	0.65
Pancreatic cancer	15.6 (8.5–34.5)	10.5 (0.8–46.9)	0.66

**Causes of death**			
**Cancer-related mortality rate**	**17**	**19**	**0.2**
Colon cancer	6	6	-
Gastric cancer	6	8	0.22
Pancreatic cancer	5	5	-
Perioperative mortality	2	3	0.5
Others	9	3	

**Tumour progression**	**15**	**16**	

Data are mean (SD), median* (range) or number of patients, t-test, chi-squared test (fisher's exact test), for unequal distribution Mann-Whitney U test, and for Kaplan-Meier method Log Rank test were used; perioperative mortality (30 days) TRD group: stomach – myocardial infarction died on postop day 4 (n = 1), stomach – multiorganic failure died on postop day 14 (n = 1); Control group: pancreas – anastomotic leak, multiorganic failure died on day 23 (n = 1), colon – pulmonal embolism died on day 2 (n = 1), stomach – pneumonia died on day 23 (n = 1); follow-up: time to last examination or death

### Follow-up studies

**Figure 4:**
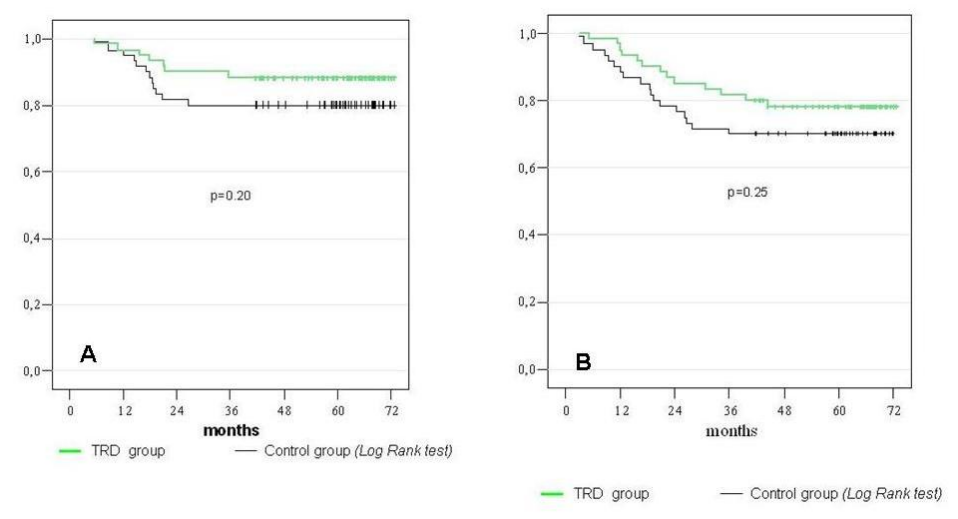
**Survival A)Overall local recurrence free rate b) Overall metastases free rate**.

**Figure 5:**
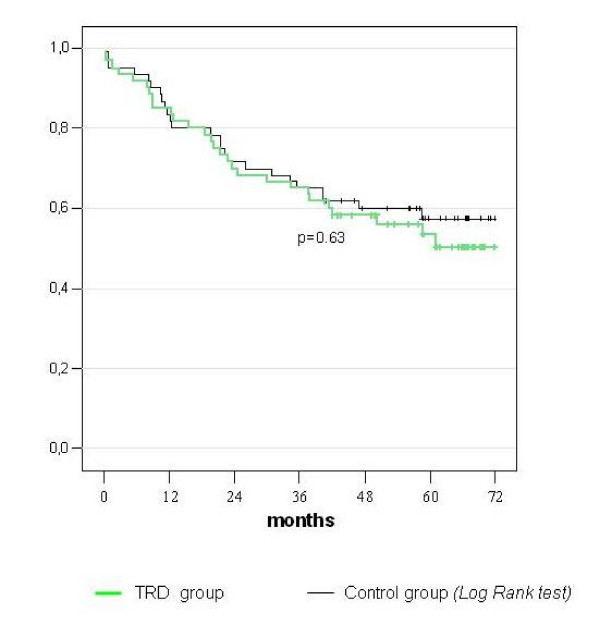
**Overall survival**.

All enrolled patients were followed-up postoperatively according to a standardized postoperative surveillance protocol. Patients receiving adjuvant chemotherapy in the TRD group did not differ from the control group with the exception of patients with gastric cancer (Table 5). The loco-regional recurrence rate and the distant metastasis rate were both minimal lower (but not statistically significant) in the TRD group as compared to the control group-despite the fact that much more patients with gastric cancer in the control group (n = 7) had received chemotherapy vs. 1 patient in TRD group (Figure 4a and 4b). Similar differences were observed in the time to local recurrences, with patients in TRD group showing distant metastatic diseases at a slightly later time point than the control group. Finally the overall mortality rate was similar in both groups. The overall survival rate and the total disease-free time are shown in figure 5.

## Discussion

Although extensively investigated over the previous years, the exact mechanisms of local or distant metastatic tumor growth, even after a R0 tumor resection, remain unclear. Cytokines produced by cells of the immune system and other relevant tissues act as mediators of immune reactions. IL-1β, IL-6, and IL-10 responses have been exhaustively investigated, particularly in their connection with tumor growth and metastatic spread in colon cancer resection [15]. Of particular interest in this setting is IL-1, a pleiotropic cytokine with numerous roles in both physiological and pathological states which is primarily produced by intraperitoneal macrophages (and not by the tumor cells). This mediator seems to be a precious indicative factor for perioperative intraabdominal tumor growth stimulation independent of the tumor type. Therefore, the IL-1beta level of gastric, colonic and pancreatic tumors was determined. It is known to be up regulated in many tumor types and has been implicated as a factor in tumor progression via the expression of metastatic and angiogenic genes and growth factors. IL-1 is mainly produced by intraabdominal macrophages in immunoactive tissues. It is a pluripotent cytokine responsible for normal physiological roles ranging from the induction of vascular permeability and fever during sepsis to the increased secretion of additional cytokines in autoimmune diseases. Therefore, an important balance exists between the beneficial and harmful effects of IL-1. Cancer cells directly produce IL-1 or can induce cells within the tumor microenvironment to do so; studies have documented constitutive IL-1alpha protein production in human and animal cancer cell lines including sarcomas and ovarian and transitional cell carcinomas. The exact mechanisms by which IL-1 promotes tumor growth remain unclear, though the protein is believed to act primarily indirectly. IL-1 induces expression of metastatic genes such as matrix metalloproteinases (MMP) and stimulates nearby cells to produce angiogenicproteins and growth factors such as VEGF, IL-8, IL-6, TNFalpha, and tumor growth factor beta (TGFalpha) [16]. Recent studies have determined the necessity of IL-1 in tumor growth, metastasis, and angiogenesis [17]. The ability of IL-1 to induce the expression of angiogenic factors such as VEGF and IL-8 is believed to promote tumor growth and metastasis. These studies highlighted the importance of IL-1 alpha in cell adhesion and invasion into extracellular matrix proteins, and studies by Voronov et al. complement findings regarding the importance of IL-1 in metastasis. They demonstrated that IL-1alpha/IL-1 knockout mice failed to develop solid tumors following injection of melanoma cells and exhibited significantly improved survival compared to the wild-type animals, which died due to lung metastases.

Meanwhile previous studies confirmed a significant association between the IL-1 polymorphisms and increased risk for tumor development in patients with intestinal type or diffuse gastric carcinoma with an odds ratio of 2.7 for carriers of IL-1 (gene 1B-511T) [18]. Barber et al. found that patients with pancreatic cancer who were homozygous for allele 2 of the IL-1 gene had significantly shorter survival rates than other groups associated with a higher CRP level [19].

The above-mentioned results on the basic influence of IL-1β on carcinogenesis do support our main end-point and demonstrate the importance of our observations concerning the significant inhibition of IL-1β production in patients with colon, gastric, and pancreatic malignancies treated with taurolidine. These results highlight the potential anti-tumor effects of TRD through a cytokine modulating effect. The major outcome criterion of our study was the influence of TRD and povidone-iodine administration on the pro-inflammatory cytokine IL-1β. Although it has been demonstrated that Povidone-iodine has to be avoided for peritoneal irrigation in peritonitis and during colonic/rectal resection, it is still in use in many german and european hospitals. This substance has been shown to be an irritant and to damage macrophages, and it is no longer used as a peritoneal irrigant in the vast majority of operations. But, this study was performed 7 years ago. Although we did notice a reduction of intraperitoneal IL-1β production after identical dilution of intraperitoneal fluid compared to PVP-iodine in the TRD group (p < 0.001), the overall disease-free time and the survival rate between the two groups were not significant. The differences in cytokine levels in peritoneal fluid are different. It is very difficult for us to explain the findings and what that means for cancer disease. Previous studies were able to detect IL-1β in the serum [20], however, in our assays we did not. This could be attributed to the lower sensitivity of the kit used. In our study the serum blood counts (leukocytes) and the CRP value have not been affected by TRD. The immunological status of the patients was evaluated from the monocyte HLA-DR status. Significantly higher values were detected on the first and second postoperative day in the TRD-group vs. povidone-iodine. This result could be interpreted as an increased immune response to TRD stimulation after surgery. Similar immunostimulant effects of TRD have been reported by other authors [21].

The fate of local tumor cell spread at the time of resection and whether it reaches the systemic circulation depends on the immunological status, the biologic behaviour of the neoplasm, and the number of the cells. In our study, intraperitoneal fluid cytology revealed no tumor cells immediately after the abdominal cavity was entered, but it was positive in one patient (out of 60) per group after tumor resection. These data are consistent with other observations [22] when resection is strictly performed according to oncological and surgical principles in curative cancer. It is known that tumor cell spillage can lead to loco-regional recurrences. Therefore, the instillation with an antitumorigenic substance like TRD is an alternative option for a complimentary perioperative treatment.

In our study of the colon cancer group, the median of the time to metastasis was 23.7 and 19.3 months in the TRD and the control group, respectively (p = 0.28). The overall survival was 35.5 months in the TRD group and 22.1 months in the control group (p = 0.34). Although not statistically significant, survival seems to be slightly better in the TRD group despite the fact that this group included patients with a higher tumor stage (UICC) and more comorbidities (in ASA scale) than the control group. A literature-based meta-analysis from the American Society of Clinical Oncology found no evidence for statistically significant survival benefit of adjuvant chemotherapy for stage I/II patients [23]. In our series there were no differences in the usage of adjuvant chemotherapy in patients with colon cancer.

The Dutch trial and the British Medical Research Cancer trial are two large prospective randomized studies evaluating the D1 versus D2 extended gastric cancer resection and lymphadenectomy [24,25]. No study showed a benefit for the D2-resection. The standard procedure performed in our participating centres was a D1-resection. Although a beneficial effect of adjuvant therapy after R0 resection is currently controversial [26,27], one patient in the TRD group and seven patients in the control group received chemotherapy (p = 0.02). This could be due to a slightly higher tumor stage in the control group. Adjuvant radiotherapy is a rare option and was only performed in UICC IIIa and UICC II in the TRD group and control group, respectively. The median of the time to metastasis was 16.5 and 14 months in the TRD and the control group, respectively (p = 0.76). The overall survival was 12.8 and 12.4 months in both groups (p = 0.65). Although not statistically significant, survival seems to be slightly better in the TRD group.

Curative resection for pancreatic cancer is possible in only 10% to 15% of cases [28], with an overall 5-year survival of 15% to 21% in most studies. In our study two patients in the TRD group (UICC stage II and III) and four patients in the control group (one patient UICC stage I, two patient UICC II, and one patient UICC III) received adjuvant chemotherapy. Although overall prognosis depends on clinicopathological staging, tumor biological features and molecular genetics, the local recurrence and metastasis rate did not differ between our groups, with the time to metastasize slightly longer in the TRD group. The median of the time to metastasis was 22.3 and 12.3 months in the TRD and the control groups, respectively (p = 0.23). The overall survival was 15.6 and 10.5 months in both groups (p = 0.66).

Povidone-iodine lavage is commonly used abdominal disinfectant. In our study the subcutaneous wound infection rate was higher in the povidone-iodine group compared to the TRD group. These findings are consistent with other results showing no benefit in povidone-iodine use for prevention of wound infection [29].

## Conclusion

In summary the basic aspects of TRD in patients with gastrointestinal cancer are as follows: The agent seems to be a strong local inhibitor of IL-1β, although its clinical influence is still unclear. TRD does not substitute surgical resection. It should be rather seen as an additional mean against metastatic tumor growth following the resection of the primary solid tumor. The interpretation of these results in terms of the antitumorigenic effects are currently being investigated in a larger German multicentre clinical prospective randomized controlled trial.

## Competing interests

Christoph A. Jacobi and Chris Braumann received support from Hoechst Marion Roussel, Germany. The company markets the agent Taurolin^® ^used as an antimicrobial substance in Germany. Rest of the authors have no competing interest to declare.

## Authors' contributions

CB designed the protocol, monitored the patients, collected, analysed and interpreted the data, and wrote the manuscript. CNG and JS recruited the patients and coordinated the study in the other two surgical centres. CM and WW monitored the patients and collected the data in the study coordinating center Berlin. JMM contributed to the idea and supported the study. CAJ contributed to the idea, designed the protocol, conceived and coordinated the study, enrolled the patients, gathered the data, analysed the data and reviewed the manuscript. CAJ and CB are guarantors of the report. All authors read and approved the manuscript.
